# Uromodulin and microRNAs in Kidney Transplantation—Association with Kidney Graft Function

**DOI:** 10.3390/ijms21165592

**Published:** 2020-08-05

**Authors:** Špela Borštnar, Željka Večerić-Haler, Emanuela Boštjančič, Živa Pipan Tkalec, Damjan Kovač, Jelka Lindič, Nika Kojc

**Affiliations:** 1 Department of Nephrology, University Medical Centre Ljubljana, Zaloška 7, 1000 Ljubljana, Slovenia; spela.borstnar@kclj.si (Š.B.); zeljka.veceric@gmail.com (Ž.V.-H.); damjan.kovac@kclj.si (D.K.); jelka.lindic@kclj.si (J.L.); 2 Faculty of Medicine, University of Ljubljana, Vrazov trg 2, 1000 Ljubljana, Slovenia; 3 Institute of Pathology, Faculty of Medicine, University of Ljubljana, Korytkova 2, 1000 Ljubljana, Slovenia; emanuela.bostjancic@mf.uni-lj.si (E.B.); ziva.pipan-tkalec@mf.uni-lj.si (Ž.P.T.)

**Keywords:** microRNA, uromodulin, kidney graft function, biomarker, kidney transplantation

## Abstract

Uromodulin and microRNAs (miRNAs) have recently been investigated as potential biomarkers for kidney graft associated pathology and outcome, with a special focus on biomarkers indicating specific disease processes and kidney graft survival. The study’s aim was to determine whether expression of serum uromodulin concentration and selected miRNAs might be related to renal function in kidney transplant recipients (KTRs). The uromodulin concentration and expression of six selected miRNAs (*miR-29c*, *miR-126*, *miR-146a*, *miR-150*, *miR-155*, and *miR-223*) were determined in the serum of 100 KTRs with stable graft function and chronic kidney disease of all five stages. Kidney graft function was estimated with routine parameters (creatinine, urea, cystatin C, and Chronic Kidney Disease Epidemiology Collaboration study equations) and precisely measured using chromium-51 labelled ethylenediaminetetraacetic-acid clearance. The selected miRNAs were shown to be independent of kidney graft function, indicating their potential as biomarkers of associated kidney graft disease processes. In contrast, the serum uromodulin level depended entirely on kidney graft function and thus reflected functioning tubules rather than any specific kidney graft injury. However, decreased concentrations of serum uromodulin can be observed in the early course of tubulointerstitial injury, thereby suggesting its useful role as an accurate, noninvasive biomarker of early (subclinical) kidney graft injury.

## 1. Introduction

Serum creatinine, urea, cystatin C, and estimation of glomerular filtration rate (GFR) via different equations are currently routinely used biomarkers of kidney graft function in clinical transplantation. Although they are characterized by low cost and rapid accessibility of results, these biomarkers are significantly less sensitive and specific than the aggressive and time-consuming gold standard, i.e., the measurement of GFR by an exogenous marker, such as chromium-51-ethylenediaminetetraacetic acid (^51^CrEDTA). Many new candidate biomarkers in kidney transplantation have been proposed and tested in recent years, which address specific pathologic processes and not merely glomerular, tubular, or overall kidney graft function. Uromodulin (also known as Tamm–Horsfall’s protein) is a urinary mucoprotein that is synthesized only in the thick ascending limb of Henle’s loop and early distal convoluted tubules of the kidneys [1]. In addition to this classical tubular secretion, to a minor degree uromodulin also sorts to the basolateral pole of tubular epithelial cells, as shown by its presence in circulation [2]. The reduced number of tubular cells seen in chronic kidney disease (CKD) due to interstitial fibrosis/tubular atrophy (IF/TA) is paralleled by the reduced urinary and serum concentrations of uromodulin [3,4,5]. The potential utility of serum [4,6,7] and urine [8] uromodulin measurement in kidney transplant recipients (KTRs) has been studied, showing an association of lower serum uromodulin levels with progression to end-stage renal disease and graft failure. Although normative ranges for serum/plasma uromodulin concentration were established over 30 years ago, its characteristics have not yet been sufficiently identified as a priority in certain instances, resulting in a failure to fully implement uromodulin in clinical practice.

MicroRNAs (miRNAs) are short, endogenous non-coding ribonucleic acids (RNAs) involved in the modulation of gene expression, mainly by inhibition of messenger RNA translation [9,10]. Recent studies have indicated an association of miRNAs with pathological processes following kidney transplantation, such as T-cell or antibody-mediated rejection, delayed graft function, and IF/TA [9,10,11]. The diagnostic accuracy of such molecules as biomarkers is still questionable, since many of them emerge on the vascular side of the glomerular filtration barrier and can therefore reflect glomerular filtration rather than a specific disease process. We have focused on searching for miRNAs that were among the most studied in the context of fibrosis (anti-fibrotic *miR-29c*) [12], endothelial dysfunction (*miR-126*) [13,14,15], and immune response (*miR-146a*) [16,17], or might even be involved in more than one physiological and/or pathogenetic process, e.g., *miR-150* [18,19], *miR-155* [16,17], and *miR-223* [20,21,22]. Moreover, our previous pilot research on miRNA association with certain most common kidney graft pathologies, such as kidney graft rejection and the recurrence of primary glomerular disease, offered interesting insights into a possible connection of selected miRNAs with underlying kidney graft pathology. For details, see also Supplementary Table S1 and Figure S1.

In this study, we investigated the association of serum uromodulin concentration (s-Uromodulin) (which emerges on the urinary side of the filtration barrier) and selected miRNAs (which emerge on the vascular side of the glomerular filtration barrier) with standard biomarkers of kidney graft function, including measurement of ^51^CrEDTA clearance. The study’s aim was firstly to investigate whether any of the proposed biomarkers are associated with the glomerular filtration and renal function in KTRs. Based on these results, the proposed biomarkers could or could not be a reliable indicator of kidney graft associated disease processes. The possible association of reliable biomarker(s) with the course of the associated disease process and kidney graft outcome is a long-term aim of this study protocol.

## 2. Results

### 2.1. Characteristics of the Study Population

**Figure 1:**
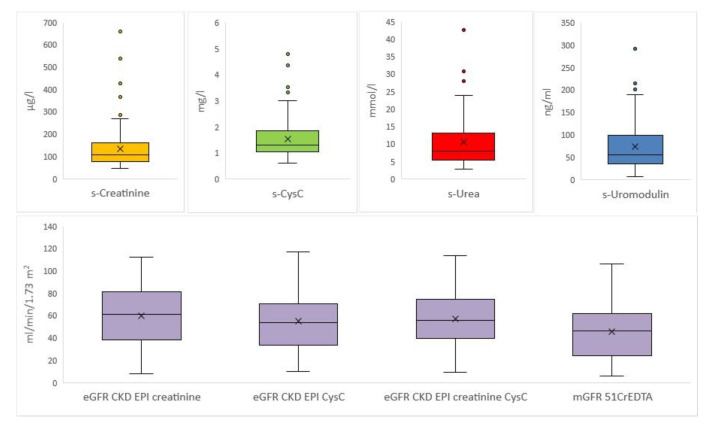
Boxplots for each parameter of glomerular filtration rate. Boxplots for serum creatinine concentration (s-Creatinine), serum cystatin C concentration (s-CysC), serum urea concentration (s-Urea), serum uromodulin concentration (s-Uromodulin), estimated glomerular filtration rate with Chronic Kidney Disease Epidemiology Collaboration study formula with s-Creatinine (eGFR CKD EPI creatinine), estimated glomerular filtration rate with Chronic Kidney Disease Epidemiology Collaboration study formula with s-CyC (eGFR CKD EPI CysC), estimated glomerular filtration rate with Chronic Kidney Disease Epidemiology Collaboration study formula with s-Creatinine and s-CysC (eGFR CKD EPI creatinine CysC), and measured glomerular filtration rate with Chromium-51-ethylenediaminetetraacetic acid clearance (mGFR ^51^CrEDTA).

The study included 100 KTRs, all Caucasian, 55 men and 45 women. The mean age was 55 ± 11 years (range 19 to 79 years). The average time from transplantation was 10 ± 7 years (range 2 to 28 years). The cohort included in the analysis had chronic kidney disease of transplanted kidney (CKD-T) of all five stages, including patients just before starting renal replacement therapy. The data presenting parameters of GFR are shown in Figure 1.

For investigation of uromodulin, the study included also 15 patients with non-kidney diseases, all Caucasian, 7 men and 8 women. The mean age was 43 ± 13 years (range 20 to 58 years).

### 2.2. Relation between Kidney Function Parameters and s-Uromodulin

**Figure 2:**
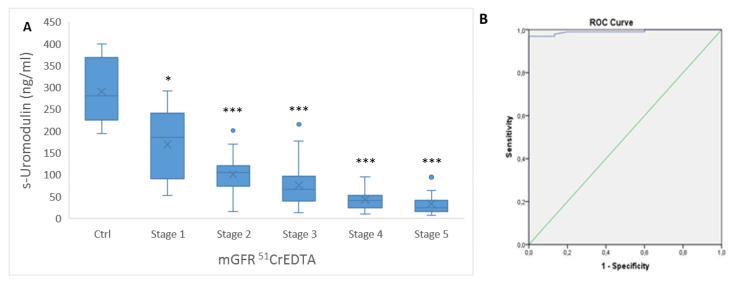
Uromodulin concentration in serum. (**A**) The boxplots for serum uromodulin concentration (s-Uromodulin) in chronic kidney disease of transplanted kidney (CKD-T) stages 1–5 based on measured GFR with ^51^CrEDTA (mGFR ^51^CrEDTA) (stage 1, *n* = 5; stage 2, *n* = 21; stage 3, *n* = 43; stage 4, *n* = 17; and stage 5, *n* = 11, with mGFR ^51^CrEDTA not possible to perform for 3 patients) in comparison to the control group (Ctrl); (**B**) ROC curve and AUC distinguishing CKD-T from the control group without any renal diseases. Legend: * *p* < 0.05, *** *p* < 0.001 (Ctrl versus Stage 1–5).

In the control group, the mean level of s-Uromodulin was 291 ± 71 ng/mL. S-Uromodulin levels decreased significantly in the group of KTRs (*p* < 0.001), in which the mean s-Uromodulin was 74 ± 53 ng/mL. We further analyzed s-Uromodulin in different stages of CKD-T (1–5, based on measured GFR with ^51^CrEDTA (mGFR ^51^CrEDTA)), in relation to the s-Uromodulin in the control group. We found that already in stage 1 of CKD-T, the s-Uromodulin significantly dropped compared to the control group (*p* = 0.013). In the other four stages (2–4), the significance of reduced s-Uromodulin compared to that in the control group was even lower (*p* < 0.001). Between CKD-T stage 1 and 2, the drop of s-Uromodulin showed borderline significance (*p* = 0.067), while s-Uromodulin was significantly lower in CKD-T stage 2 compared to CKD-T stage 3 (*p* < 0.05) and in CKD-T stage 3 compared to CKD-T stage 4 (*p* < 0.05). There were no significant differences between stages CKD-T 4 and 5 (Figure 2A).

According to receiver operating characteristics (ROC), the area under the curve (AUC) represents the probability that a randomly selected patient will have a lower or higher test result than a randomly selected control. The ROC curve of s-Uromodulin in KTRs demonstrated an AUC of 0.991 (SE-0.007) (95% Cl 0.977–1.00, *p* < 0.001) at an optimal cut-off of 191.5 ng/mL with 97% sensitivity and 100% specificity (Figure 2B).

Analyzing bivariate correlations using Spearman’s correlation coefficient, s-Uromodulin was significantly associated with all parameters of kidney graft function. The highest correlation coefficient was noted for estimated glomerular filtration rate (eGFR) with Chronic Kidney Disease Epidemiology Collaboration study formula with serum creatinine concentration (s-Creatinine) and serum cystatin C concentration (s-CysC) (eGFR CKD EPI creatinine CysC) (Rho = 0.758, *p* < 0.001), followed by serum urea concentration (s-Urea) (Rho = −0.740, *p* < 0.001), eGFR with Chronic Kidney Disease Epidemiology Collaboration study formula with s-Creatinine (eGFR CKD EPI creatinine) (Rho = −0.736, *p* < 0.001), s-CysC (Rho = −0.720, *p* < 0.001), eGFR with Chronic Kidney Disease Epidemiology Collaboration study formula with s-CysC (eGFR CKD EPI CysC) (Rho = 0.718, *p* < 0.001), s-Creatinine (Rho = −0.698, *p* < 0.001), and mGFR ^51^CrEDTA (Rho = 0.669, *p* < 0.001) (Table 1).

### 2.3. Relation between Kidney Function Parameters and Expression of Selected miRNAs

**Figure 3:**
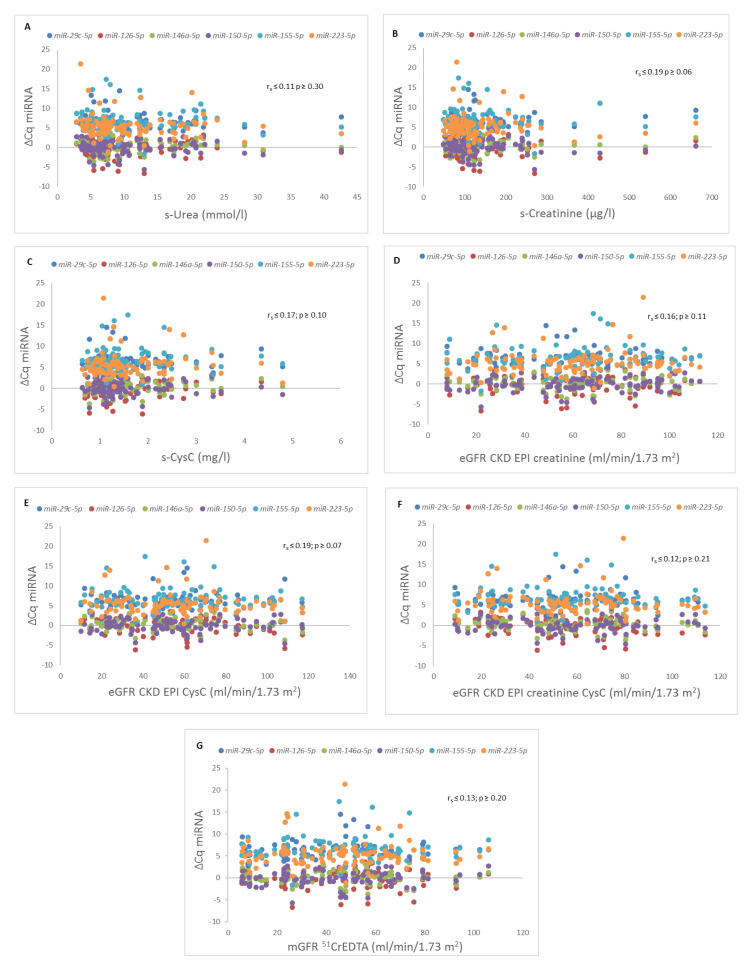
Correlations between different microRNAs (miRNAs) and parameters of kidney graft function. Correlation between different miRNAs and (**A**) serum urea concentration (s-Urea), (**B**) serum creatinine concentration (s-Creatinine), (**C**) serum cystatin C concentration (s-CysC), (**D**) estimated glomerular filtration rate with Chronic Kidney Disease Epidemiology Collaboration study formula with s-Creatinine (eGFR CKD EPI creatinine), (**E**) estimated glomerular filtration rate with Chronic Kidney Disease Epidemiology Collaboration study formula with s-CysC (eGFR CKD EPI CysC), (**F**) estimated glomerular filtration rate with Chronic Kidney Disease Epidemiology Collaboration study formula with s-Creatinine and s-CysC (eGFR CKD EPI creatinine CysC), (**G**) measured glomerular filtration rate with Chromium-51-ethylenediaminetetraacetic acid clearance (mGFR ^51^CrEDTA).

While *miR-126*, *miR-146a*, and *miR-150* were expressed in all 100 samples of serum, *miR-29c*, *miR-155*, and *miR-223* were not expressed in 12, 8, and 8 serum samples, respectively. Using the Pearson correlation coefficient, none of the six analyzed miRNAs significantly correlated with the parameters of kidney graft function (Figure 3).

## 3. Discussion

Although extensive scientific effort has been focused on developing biomarkers to detect kidney allograft disease processes such as rejection or IF/TA, few assays have moved from the research arena to clinical routine. The obstacle to the successful initiation of their clinical use is the still insufficiently validated specificity for renal pathology. Similar to other molecules and proteins, these biomarkers are also subject to glomerular filtration, tubular secretion, and reabsorption. Many of them, depending on the site of their production and paths of elimination, probably reflect merely kidney function and not disease. The identification of sensitive biomarkers able to reflect the subclinical steps of a pathologic process, such as rejection, is therefore of the utmost relevance to recipients of kidney transplants [23,24,25].

Our research confirmed previous reports showing significantly higher s-Uromodulin in healthy controls (patients without kidney disease) compared to KTRs, who in general belong to the group of CKD patients [4,5,26]. In line with the findings of other similar research in patients with kidney transplants [6], s-Uromodulin levels in our KTRs decreased stepwise from those with almost preserved graft function to the lowest values in KTRs with pre-dialysis CKD-T. We also showed in this study that s-Uromodulin correlates with all parameters of kidney graft function, the strongest association being observed for eGFR CKD EPI creatinine CysC, followed by the serum level of urea, eGFR CKD EPI creatinine, serum level of CysC, eGFR CKD EPI CysC, serum level of creatinine, and ^51^CrEDTA. Decreased s-Uromodulin was observed in the earliest stages of CKD-T when other markers, such as serum creatinine and even the precise method of GFR determination with ^51^CrEDTA, had still not crossed the reference range. This indicates that reabsorption of uromodulin, which is exclusively a product of tubules, is probably compromised from the earliest stages of tubulo-interstitial injury. Reduced s-Uromodulin may therefore be a more sensitive indicator of early kidney graft dysfunction not detected by serum glomerular filtration markers. In view of the simple routine of s-Uromodulin measurement and its low costs, monitoring the dynamics of s-Uromodulin concentration may serve as an accurate, noninvasive predictive biomarker of kidney graft injury and outcome.

Given that the concentration of uromodulin strongly reflects renal function, one would assume that it would never be able to act as a reliable parameter of specific kidney graft pathology. However, exceptions have already been found in the field of specificity for certain pathologies of native kidneys, such as gout [27] or Balkan nephropathy [2]. Accordingly, in the case of kidney transplantation, s-Uromodulin could be especially useful to clinicians aware of the advantages of biomarkers reflecting subclinical tubular injury. Regular s-Uromodulin checkups in KTRs could, in our opinion, become a useful tool for early detection of subclinical processes involving tubules and interstitium (such as acute tubulointerstitial rejection), or timely surveillance of IF/TA. This assumption, however, needs further exploration.

The miRNAs investigated in the present study have so far been extensively related to various physiological and pathological states of kidney graft pathology (see also Supplementary Table S1). We did not observe any association between the circulating levels of selected miRNAs (*miR-29c*, *miR-126*, *miR-146a*, *miR-150*, *miR-155*, and *miR-223*) and kidney graft function (estimated and measured by the reference method at the same time point of miRNA analysis). Previously published studies have shown that the expression of some miRNAs, such as *miR-126* or *miR-223*, is associated with renal function in an up- or downregulated manner [11,15,28]. However, many of those studies were performed with patients with CKD without kidney transplants [15] and/or focused on the functionally unstable period of delayed graft function immediately after transplantation [11,17]. The essential advantage of our analysis is that we used the reference method for GFR measurement (^51^CrEDTA) and not only routinely used noninvasive biomarkers and/or GFR equations, but the patient cohort in this study was also larger than in most of the so far published research in the field of biomarkers in kidney transplantation [10,11,17,28].

Since selected miRNAs are independent of kidney graft function, they can be reliably used as biomarkers of various pathological processes in KTRs without adjustments to kidney function. In line with previous findings, the results of our pilot study performed on a subgroup of patients with indications of kidney graft biopsy showed a significant association with the miRNAs selected here, with histologically proven antibody-mediated kidney graft rejection and recurrence of primary glomerulonephritis. In this regard, *miR-29c* expression especially has shown potential for differentiating between these two pathologies (see also Supplementary Figure S1). Unfortunately, the sample examined was too small for reliable inference, but nevertheless pointed to the feasibility of conducting further prospective analysis with systematic serum sampling for miRNA determination and planned implementation of kidney graft biopsy, which is the long term aim of this study.

## 4. Materials and Methods

A prospective clinical study (NCT04413916) was conducted at the Department of Nephrology, University Medical Centre Ljubljana, Slovenia and the Institute of Pathology, Faculty of Medicine, University of Ljubljana. The study was approved by the National Medical Ethics Committee of the Republic of Slovenia (permit number 24k/06/12 approved on 15/06/2012 and 0120-625/2017/4 on 18/12/2017 (revised version number 0120-625/2017/11 on 12/12/2019)) and conducted in accordance with the Declaration of Helsinki. All patients signed informed consent.

### 4.1. Study Population Inclusion and Exclusion Criteria

The study included 100 kidney transplant recipients from the Slovenian Center for Kidney Transplantation. Inclusion criteria were stable graft function for more than 3 months (changes in creatinine concentration < 20%) and a time of transplantation at least two years before the study entry, since we wanted to study the expression of miRNAs in stable conditions and eliminate the influence of recent transplantation, replacement therapy, and rapid changes in renal function. The subsequent data analysis revealed unstable graft function in only one patient. Exclusion criteria were an age less than 18 years, symptomatic heart failure, malignancy, pregnancy or lactation, newly introduced drugs that may affect the function of the graft, and treatment with trimethoprim-sulfamethoxazole or cimetidine. As a control group, 15 patients with non-kidney diseases were included (nonspecific skin lesions *n* = 13, erosive stomatitis *n* = 1, and paraneoplastic dermatitis *n* = 1).

### 4.2. Measurement of Serum Creatinine, Serum Urea, and Cystatin C Concentration

Blood sampling was performed on the same day as the measurement of GFR ^51^CrEDTA clearance, but before the injection of ^51^CrEDTA. S-Creatinine was measured with the kinetic colorimetric compensated Jaffe assay (Siemens Healthcare Diagnostics Inc., Tarrytown, NY, USA) and with a calibrator traceable to primary reference material with values assigned by isotope dilution mass spectrometry [29]. S-CysC was measured using the particle-enhanced immunonephelometric method (Siemens Healthcare Diagnostics, Marburg, Germany) [30]. S-urea was determined by a Roch-Ramel enzyme reaction with urease and glutamate dehydrogenase (Siemens Healthcare Diagnostics Inc., Tarrytown, NY, USA).

### 4.3. eGFR

eGFR was calculated using Chronic Kidney Disease Epidemiology Collaboration study formulae with s-Creatinine (eGFR CKD EPI creatinine) and s-CysC (eGFR CKD EPI CysC) or with both s-Creatinine and s-CysC (eGFR CKD EPI creatinine CysC) [31].

### 4.4. Measurement of ^51^CrEDTA Clearance

mGFR ^51^CrEDTA was determined from a single ^51^CrEDTA injection (activity 3 MBq) and four blood samples taken 120, 180, 240, and 300 min after intravenous application of the marker according to British Nuclear Medicine Society guidelines, using the By weight method. Samples were measured using a gamma counter (Hidex, Turku, Finland); mGFR ^51^CrEDTA was calculated and then adjusted to the patient’s body surface area (Haycock formula) and specified as mL/min/1.73 m^2^ [32].

### 4.5. Measurement of s-Uromodulin

S-Uromodulin was assessed in 20 controls and in 100 KTRs. All serum samples were stored at –80 °C before measurements were performed. Measurements of s-Uromodulin were performed using commercial ELISA (Euroimmun, Medizinische Labordiagnostika AG, Lübeck, Germany), as described previously, based on the manufacturer’s instructions [5]. Characteristics of the ELISA were as follows, given by the manufacturer: detection limit for plasma samples 2 ng/mL; mean linearity recovery 97% (83–107% at 59–397  ng/mL); intra-assay precision 1.8–3.2% (at 30–214  ng/mL); inter-assay precision 6.6–7.8% (at 35–228  ng/mL); and inter-lot precision 7.2–10.1% (at 37–227  ng/mL). Data analysis was performed using the program Analysis Software Gen5 (Gen5 2.09, BioTek).

### 4.6. miRNA Quantification

Total RNA isolation was performed using 200 µL of serum and a miRNeasy serum/plasma advanced kit (Qiagen, Hilden, Germany) according to the manufacturer’s protocol. Elution was performed using 20 µL of RNAse-free water. The successful isolation procedure was confirmed by adding spike-ins and subsequent quantification of these spike-ins.

miRNAs *miR-29c*, *miR-126*, *miR-146a*, *miR-150*, *miR-155*, and *miR-223* were analyzed using the miRCURY LNA miRNA PCR system (Qiagen, Hilden, Germany). As the reference genes, *miR-103a-3p*, *miR-191*, and *miR-423* were used according to the manufacturer’s instruction. The possibility of hemolysis was excluded by quantifying *miR-23a* and *miR-451a*. All the reagents were from Qiagen, except where otherwise indicated. qPCR was carried out using Rotor Gene Q.

For reverse transcription, a miRCURY LNA RT Kit was used in a 10 μL reaction master mix containing 2 µL of total RNA, according to the manufacturer’s instructions. The resulting reverse transcription was diluted 20-fold and 3 μL was used in a 10 μL reaction master mix, according to the manufacturer’s instructions. All the qPCR reactions were performed in duplicate. Prior to qPCR, RNA samples were pooled, followed by RT and qPCR as described above. Efficiency was tested for each analyzed miRNA using 10-fold dilutions and qPCR was performed in triplicate. The signal was collected at the endpoint of every cycle. Following amplification, melting curve analysis of PCR products was performed to verify the specificity and identity. Melting curves were acquired on the SYBR channel using a ramping rate of 0.7 °C/60 s for 60–95 °C.

### 4.7. Statistical Analysis

Correlations were analyzed using Spearman’s rank correlation coefficient (Spearman’s rho) or the Pearson correlation coefficient. To present relative gene expression, ΔCq was calculated for miRNA expression [33]. The *t*-test was used to calculate the difference between the expression of uromodulin between the control group and the kidney transplant group. For all statistical analyses, SPSS analytical software (IBM SPSS statistics, version 24.0, Armonk, NY, USA) was used with a cut-off point at *p* < 0.05.

## 5. Conclusions

In the present study, we evaluated six selected miRNAs (*miR-29c*, *miR-126*, *miR-146a*, *miR-150*, *miR-155*, and *miR-223*) and s-Uromodulin as biomarkers of kidney function in KTRs. The selected miRNAs and s-Uromodulin were compared not only with conventional GFR biomarkers (creatinine, cystatin, and estimated GFR), but also, as a novelty, with the radioisotope method, providing significant reinforcement to the credibility of the findings. To the best of our knowledge, this is the first study to compare these markers with the clinical gold standard of GFR (i.e., ^51^CrEDTA clearance) in KTRs.

In brief, the selected miRNAs are independent of kidney graft function, indicating their potential as biomarkers of associated etiopathogenesis of kidney graft disease processes. In contrast, s-Uromodulin is dependent on all observed parameters of kidney graft function. However, s-Uromodulin reliably reflects the early stages of kidney graft disfunction, in which conventional GFR-related biomarkers, including ^51^Cr EDTA, are still within normal limits. Since uromodulin is synthetized exclusively within tubules, though it is not a disease-specific marker, it can point to a predominantly tubular injury. Further research is needed to explore the timely expression of uromodulin and miRNAs associated with transplant pathology patterns, in order to detect kidney allograft pathology on a subclinical level, tailor response to therapy, and predict graft outcome.

## Figures and Tables

**Table 1:** Bivariate correlations between serum uromodulin concentration (s-Uromodulin) and parameters of kidney graft function: serum creatinine concentration (s-Creatinine), serum cystatin C concentration (s-CysC), serum urea concentration (s-Urea), estimated glomerular filtration rate with Chronic Kidney Disease Epidemiology Collaboration study formula with s-Creatinine (eGFR CKD EPI creatinine), estimated glomerular filtration rate with Chronic Kidney Disease Epidemiology Collaboration study formula with s-CysC (eGFR CKD EPI CysC), estimated glomerular filtration rate with Chronic Kidney Disease Epidemiology Collaboration study formula with s-Creatinine and s-CysC (eGFR CKD EPI creatinine CysC) and measured GFR with Chromium-51-ethylenediaminetetraacetic acid (mGFR ^51^CrEDTA).

Parameters of Kidney Graft Function	Spearman Correlation to s-Uromodulin	*p*
s-Creatinine	−0.698	<0.001
s-CysC	−0.720	<0.001
s-Urea	−0.740	<0.001
eGFR CKD EPI creatinine	0.736	<0.001
eGFR CKD EPI CysC	0.718	<0.001
eGFR CKD EPI creatinine CysC	0.758	<0.001
mGFR ^51^CrEDTA	0.669	<0.001

## Supplementary Materials

Supplementary materials can be found at https://www.mdpi.com/1422-0067/21/16/5592/s1, Table S1: Association of selected miRNAs with kidney graft disorder and relevant gene target(s), Figure S1: Association of miRNAs (*miR-29c, miR-126, miR-146a, miR-150, miR-155, miR-223*) with kidney graft disease in patients with performed kidney graft biopsy due to indication after miRNA measurement.

## Abbreviations

CKD	chronic kidney disease
CKD-T	chronic kidney disease of transplanted kidney
^51^CrEDTA	chromium-51-ethylenediaminetetraacetic acid
GFR	glomerular filtration rate
eGFR	estimated GFR
eGFR CKD EPI creatinine	eGFR with Chronic Kidney Disease Epidemiology Collaboration study formula with s-Creatinine
eGFR CKD EPI CysC	eGFR with Chronic Kidney Disease Epidemiology Collaboration study formula with s-CysC
eGFR CKD EPI creatinine CysC	eGFR with Chronic Kidney Disease Epidemiology Collaboration study formula with s-Creatinine and s-CysC
mGFR ^51^CrEDTA	measured GFR with ^51^CrEDTA
IF/TA	interstitial fibrosis/tubular atrophy
KTRs	kidney transplant recipients
miRNA	microRNA
RNA	ribonucleic acid
s-Creatinine	serum creatinine concentration
s-CysC	serum cystatin C concentration
s-Urea	serum urea concentration
s-Uromodulin	serum uromodulin concentration
