# Supplementary material for: Uromodulin and microRNAs in Kidney Transplantation—Association with Kidney Graft Function
Source: Int J Mol Sci. 2020 Aug 5;21(16):5592. doi: 10.3390/ijms21165592 (PMC7460670; doi:10.3390/ijms21165592)
Supplement: Supplementary file 1 [file ijms-21-05592-s001.pdf]

Supplementary materials

Table S1. Association of selected miRNAs with kidney graft disorder and relevant gene target(s).

Selected miRNA	Associated Disorder	Relevant Gene Target or Downstream Molecule	Reference
miR-126	endothelial dysfunction, CKD, atherosclerosis	*VCAM, CXCL12*	[13]<br>Mondadori dos Santos A, et al. miR-126 Is Involved in Vascular Remodeling under Laminar Shear Stress. *Biomed Res Int*. 2015; 2015: 497280.<br>[15]<br>Fujii R, Yamada H, Yamazaki M, et al. Circulating microRNAs (miR-126, miR-197, and miR-223) are associated with chronic kidney disease among elderly survivors of the Great East Japan Earthquake. *BMC Nephrol*. 2019; 20(1): 474.
miR-223	glomerular endothelial cells injury, vascular calcification, kidney graft AR, AKI, CKD	*NFκB, NFIA, RHOB*	Ulbing M, Kirsch AH, Leber B, et al. MicroRNAs 223-3p and 93-5p in patients with chronic kidney disease before and after renal transplantation. *Bone*. 2017;95:115-123.<br>[21]<br>[15]<br>Fujii R, Yamada H, Yamazaki M, et al. Circulating microRNAs (miR-126, miR-197, and miR-223) are associated with chronic kidney disease among elderly survivors of the Great East Japan Earthquake. *BMC Nephrol*. 2019; 20(1): 474
miR-150	pro-fibrotic, differentially expressed between IFTA and GN, lupus GN, chronic allograft disfunction	*SOCSI*	Zhou H, Hasni SA, Perez P, et al. miR-150 promotes renal fibrosis in lupus nephritis by downregulating SOCS1. *J Am Soc Nephrol*. 2013; 24(7): 1073-1087.<br>Liu X, Fu B, Chen D, et al. miR-184 and miR-150 promote renal glomerular mesangial cell aging by targeting Rab1a and Rab31. *Exp Cell Res*. 2015; 336(2): 192-203.<br>Nakhjavani M, Etemadi J, Pourlak T, Mirhosaini Z, Zununi Vahed S, Abediazar S. Plasma levels of miR-21, miR-150, miR-423 in patients with lupus nephritis. *Iran J Kidney Dis*. 2019; 13(3): 198-206.
miR-29c	pro-fibrotic, renal fibrosis, unilateral urinary obstruction, IgAGN	*HDAC, DKK1*,collagen I, II, IV, tropomyosin 1α	Fang Y, Yu X, Liu Y, et al. miR-29c is downregulated in renal interstitial fibrosis in humans and rats and restored by HIF-α activation. *Am J Physiol Renal Physiol*. 2013; 304(10): F1274-F1282.<br>Wang G, Kwan BC, Lai FM, Chow KM, Li PK, Szeto CC. Urinary miR-21, miR-29, and miR-93: novel biomarkers of fibrosis. *Am J Nephrol*. 2012; 36(5): 412-418.<br>[12]<br>Chun-Yan L, Zi-Yi Z, Tian-Lin Y, et al. Liquid biopsy biomarkers of renal interstitial fibrosis

			based on urinary exosome. *Exp Mol Pathol*. 2018; 105(2): 223-228. [17]
miR-146a	AKI, renal cancer, kidney graft AR	*NFκB*	Wang G, Kwan BC, Lai FM, Chow KM, Li PK, Szeto CC. Elevated levels of miR-146a and miR-155 in kidney biopsy and urine from patients with IgA nephropathy. *Dis Markers*. 2011; 30(4): 171-179.<br>Ichii O, Otsuka S, Sasaki N, Namiki Y, Hashimoto Y, Kon Y. Altered expression of microRNA miR-146a correlates with the development of chronic renal inflammation. *Kidney Int*. 2012; 81(3): 280-292.<br>Amrouche L, Desbuissons G, Rabant M, et al. MicroRNA-146a in Human and Experimental Ischemic AKI: CXCL8-Dependent Mechanism of Action. *J Am Soc Nephrol*. 2017; 28(2): 479-493.
miR-155	vascular calcification, CKD, kidney graft AR	*SMAD* family, *PU.1, HDAC*	Liang J, Tang Y, Liu Z, et al. Increased expression of miR-155 correlates with abnormal allograft status in solid organ transplant patients and rat kidney transplantation model. *Life Sci*. 2019;227:51-57. [28]<br>Zununi Vahed S, Poursadegh Zonouzi A, Ghanbarian H, et al. Differential expression of circulating miR-21, miR-142-3p and miR-155 in renal transplant recipients with impaired graft function. *Int Urol Nephrol*. 2017; 49(9): 1681-1689.<br>Zhang W, Li X, Tang Y, Chen C, Jing R, Liu T. miR-155-5p Implicates in the Pathogenesis of Renal Fibrosis via Targeting SOCS1 and SOCS6. *Oxid Med Cell Longev*. 2020; 2020: 6263921.

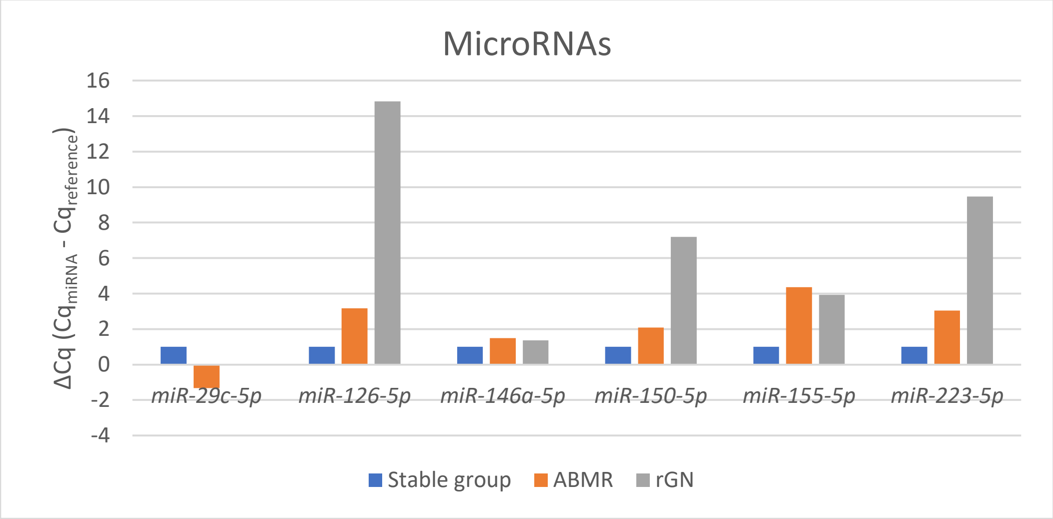

Figure S1. Association of miRNAs (*miR-29c*, *miR-126*, *miR-146a*, *miR-150*, *miR-155*, *miR-223*) with kidney graft disease in patients with performed kidney graft biopsy due to indication after miRNA measurement. Blood samples of patients with recurrent glomerulonephritis (rGN; $n = 3$) after kidney transplantation presented higher expression

of *miR-126, miR-150, miR-223* than patients with antibody mediated rejection (ABMR, *n* = 11) and patients that were without indication for biopsy (stable patients' group; *n* = 85). *miR-155* presented higher expression in both, rGN and ABMR, compared to the stable group. *miR-29c* expression distinguished between pathologies, showing a distinct pattern of expression in the setting of ABMR and rGN post transplantation. The results of the pilot study were presented as oral abstract at ASN Kidney Week, Washington DC, 2019.
